# A Rare Encounter: Anorectal Melanoma as a Cause of Rectal Bleeding

**DOI:** 10.7759/cureus.108485

**Published:** 2026-05-08

**Authors:** Eran Hapuarachchi, Pramodh Chandrasinghe, Sumudu Kumarage

**Affiliations:** 1 Department of General Surgery, National Hospital of Sri Lanka, Colombo, LKA; 2 Department of Surgery, Faculty of Medicine, University of Kelaniya, Ragama, LKA

**Keywords:** abdominoperineal resection, anorectal, anorectal melanoma, apr, melanoma, mucosal melanoma, rectal cancer

## Abstract

Anorectal melanoma is a rare malignancy. It has a poor prognosis due to delayed detection and aggressive tumor biology. In addition, there are no established guidelines for its staging or management, which further complicates the delivery of care. We present a case of an advanced anorectal melanoma in a 63-year-old male from Sri Lanka who presented with per-rectal bleeding and altered bowel habits. He underwent laparoscopic abdominoperineal resection due to the distal site and advanced stage of the tumor and was then scheduled for adjuvant chemotherapy, which he refused in favor of Ayurvedic treatment.

## Introduction

Melanomas are neoplasms arising from melanocytes. They are mainly cutaneous in origin, but around 1% of malignant melanomas are mucosal [1]. Mucosal melanomas occur most commonly in the eyes and then in the anal canal [2]. Other regions include the rectum, nasal and oral cavities up to the larynx and esophagus, and the vagina and cervix, although any mucosa may be involved. Melanomas account for only 0.05% of colorectal cancers and 1% of anal cancers [3], thus highlighting the rarity of this clinical entity. Furthermore, prognosis is poor; five-year survival for those with metastatic disease is 0%, and for those limited to local disease is 26.7% [2]. To add to its gravity, there are no proper pathological staging criteria or management guidelines. Other challenges we faced during this patient’s management were whether to perform radical versus local resection, whether inguinal lymph node sampling was needed, and what kind of adjuvant therapy was to be started.

## Case presentation

A 63-year-old male from Sri Lanka, with a past history of type 2 diabetes mellitus and a minor stroke, presented with complaints of progressively worsening fresh and painful per rectal bleeding, an increase in frequency of bowel motions, loss of weight, and generalized weakness for a duration of three months. There was no change in the consistency of his stool, no abdominal pain, loss of appetite, mucus discharge, or lower rectal symptoms. He did not have features suggestive of obstruction or distant spread. His diet was a balanced one, and he did not smoke or drink. His exercise tolerance was satisfactory, with a metabolic equivalent of task (MET) score of 4. His past surgical and allergic histories were unremarkable. There was no family history of cancer. General examination was unremarkable, and digital rectal examination revealed a hard mass at about 6 cm from the anal verge.

**Figure 1:**
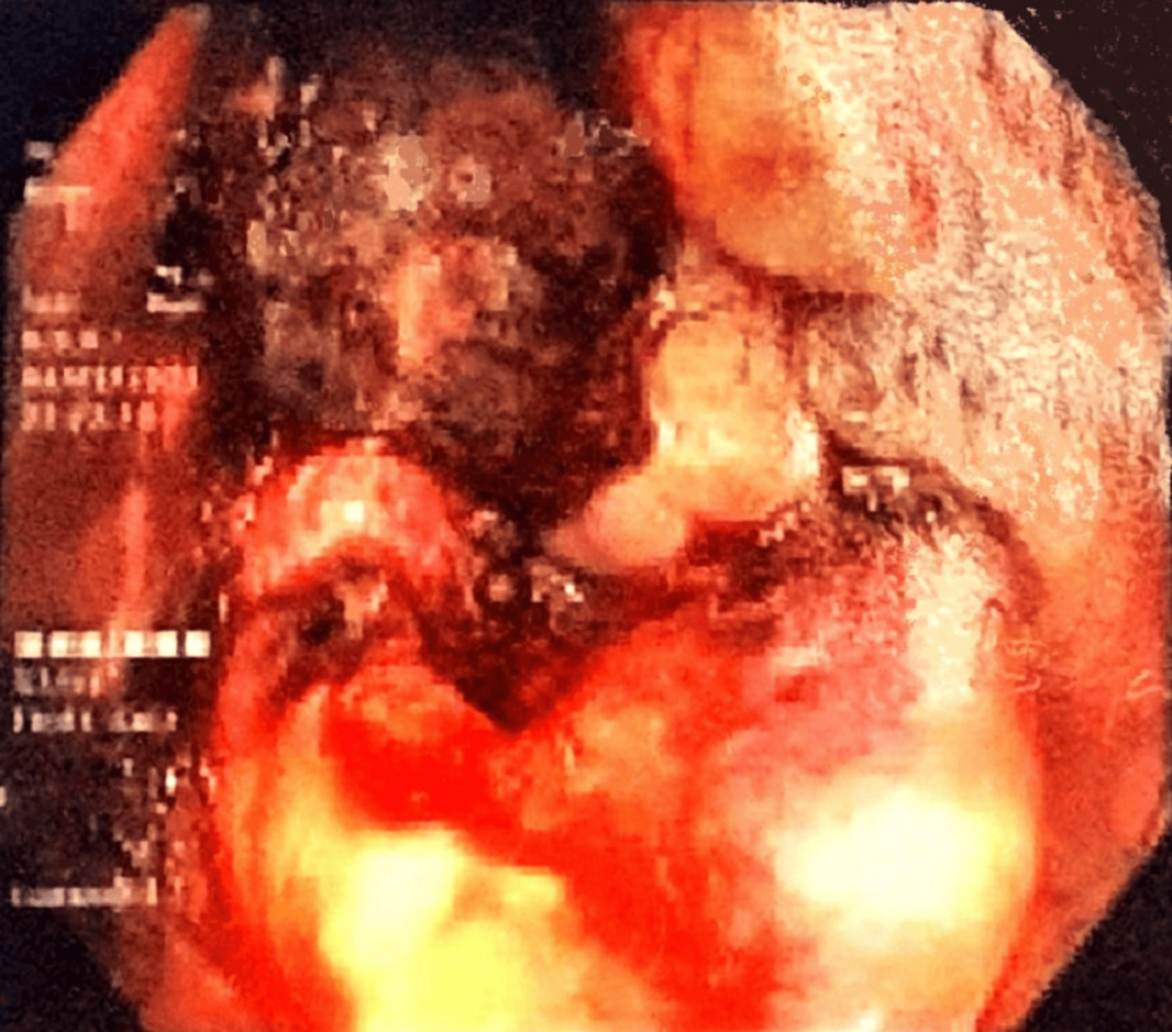
Endoscopic view of a pigmented lesion in the lower rectum.

**Figure 2:**
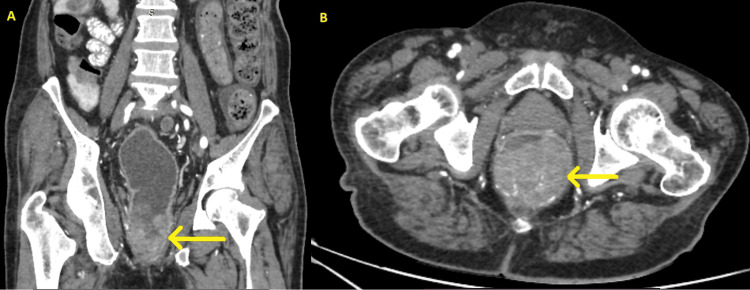
CT images of the lesion in (A) coronal and (B) axial sections.

A colonoscopy was performed, which revealed a large and irregular circumferential mass in the lower rectum without synchronous lesions. Parts of the mass were suspected to be necrotic, as they appeared black (Figure 1), and a biopsy was performed. However, histology was highly suggestive of malignant melanoma, and immunohistochemistry was required for confirmation. A staging contrast-enhanced computed tomography (CECT) of the chest, abdomen, and pelvis revealed a 6.7 cm long, irregular mass in the lower rectum (Figure 2) with mesorectal and inferior mesenteric lymph node enlargement but without distant metastasis. Carcinoembryonic antigen (CEA) was 2.36 ng/mL. By this time, around one month since the initial presentation, he had developed palpable inguinal lymph nodes, so a fine needle aspiration cytology (FNAC) was performed, which came back as reactive lymphadenitis without atypical cells. A black colored perianal lump of new onset was also noted.

**Figure 3:**
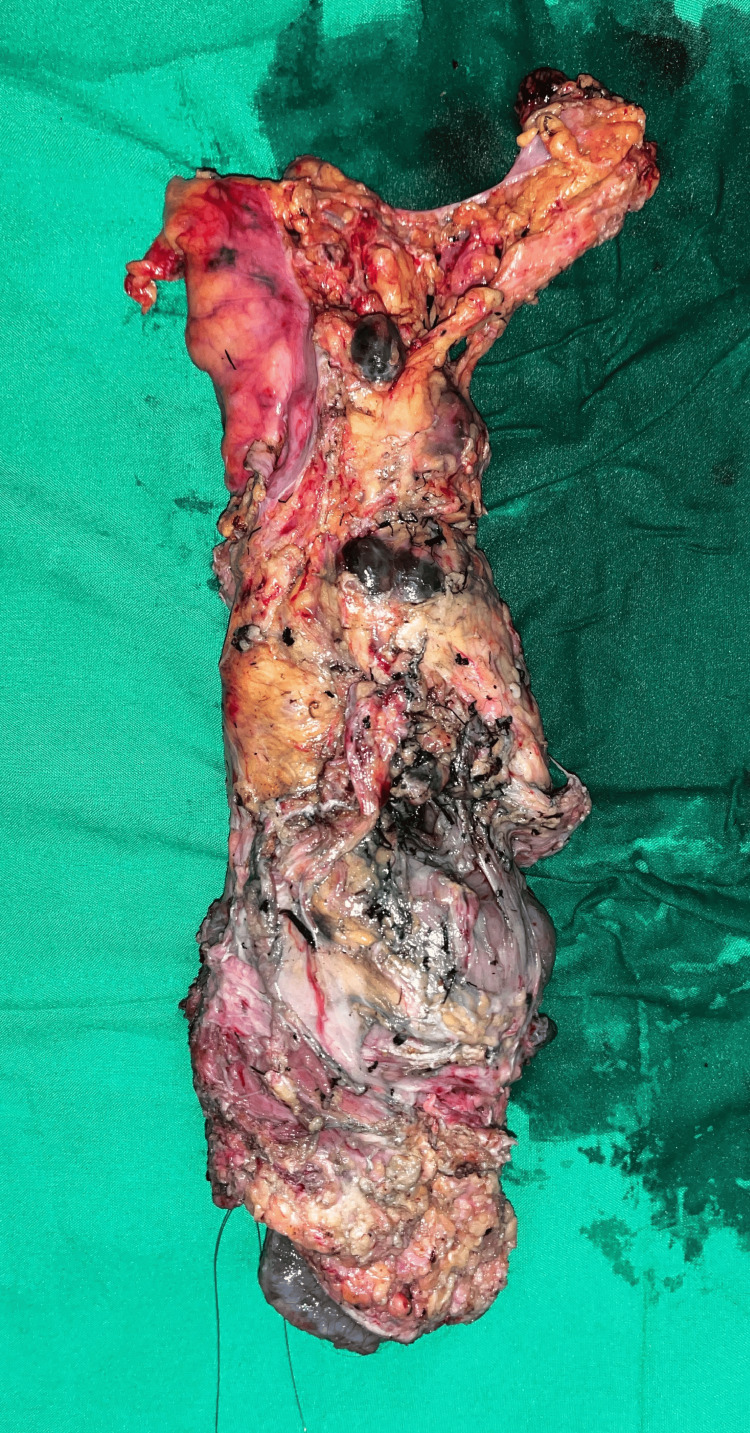
Abdominoperineal resection specimen showing pigmented lesions scattered throughout.

A multidisciplinary team meeting then ensued, involving two colorectal surgeons, an interventional radiologist, a histopathologist, an oncologist, an anesthetist, a nutritionist, a physician, a stoma nurse, the patient, and his family. It was decided to go ahead with a laparoscopic abdominoperineal resection (APR) as there was no proven place for neoadjuvant therapy. Histology confirmed a malignant anorectal mucosal melanoma. Circumferential resection margins were involved (Figure 3), but distal and proximal ends were spared. A total of 15 out of 40 lymph nodes were positive, with extranodal extension. Lymphovascular and perineural invasions were present.

The patient was discharged home on postoperative day five. He was followed up for about one month, after which he was lost to follow-up, as he refused adjuvant chemotherapy in favor of native Ayurvedic treatment.

## Discussion

Anorectal melanomas present at a median age of 55 years [2]. There are no known risk factors, but some studies show that HIV may be one, and mutations in BRAF and cKIT genes are thought to be key players in pathogenesis [3-5]. The commonest presenting complaint is per rectal bleeding, amongst other symptoms like anal pain, altered bowel habits, tenesmus, and an anal lump [2]. Presentation is late, with about 32% of patients having distant metastasis [6] and 60% having regional nodal metastasis on presentation [7]. Common sites of metastasis include inguinal, mesenteric, hypogastric, and para-aortic lymph nodes, the liver, lungs, skin, and the brain [2]. The reasons for delayed diagnosis include discrete location, similarity to benign conditions like hemorrhoids or skin tags, and the fact that about one-fifth are amelanotic subtypes [2].

On histopathological examination, four types of anorectal melanoma may be identified: epithelioid, spindle-cell, lymphoma-like, and pleomorphic anorectal melanoma. The spindle-cell variety can be easily misdiagnosed as a gastrointestinal stromal tumor, and therefore immunohistochemistry with tests for S100 (highly sensitive), HMB-45, and Melan-A should be used [8].

Staging is performed with CECT of the chest, abdomen, and pelvis to assess distant metastasis, along with MRI of the pelvis to better evaluate local spread. Brain MRI is indicated if radical resection is planned [5]. In our case, it was decided that any additional information that could be gathered from an MRI would not change the patient's management preoperatively, as there is no definite neoadjuvant therapy for anorectal melanoma, unlike in other rectal cancers, where accurate staging is needed to plan neoadjuvant therapy. Thus, compounded by limited resources, it was decided not to proceed with MRI.

Clinically or radiologically apparent inguinal lymph nodes can be sampled, and if positive, inguinal block dissection may be carried out [9]. Sentinel node biopsy or prophylactic inguinal lymphadenectomy does not seem to have a benefit in these patients [10].

There is no pathologic staging system for anorectal melanoma, but TNM (tumor, node, and metastasis) staging has been used in some reports. A clinical staging system has also been devised where stage I is clinically localized disease, stage II is regional lymph-nodal disease, and stage III is disseminated disease [8].

The mainstay of treatment is surgery. Since prognosis is poor, the aim of surgery should be to improve quality of life, i.e., achieve clear margins in the least radical fashion with spared sphincters, unless there is sphincter involvement. APR achieves better local control but is a more invasive surgery and does not offer superior outcomes to local excision [2,11].

Adjuvant radiation therapy for anorectal melanoma may improve locoregional control despite a limited impact on overall survival [2]. Adjuvant chemotherapy improved the relapse-free survival benefit in randomized trials of patients with mucosal melanoma and resected regional lymph nodes [12]. Immunotherapy also seems to have a place as an adjuvant therapy: for the uncommon patient with a BRAF V600 mutation-positive tumor (less than 10% of all mucosal melanomas), targeted therapy with dabrafenib plus trametinib is also an option [12]. None of these forms of adjuvant therapy has strong evidence supporting it, and further studies are necessary.

The prognosis of patients with anorectal melanoma is poor, with five-year survival being 26.7%, 9.8%, and 0% for stages I, II, and III, respectively [13]. However, positive prognostic factors include thickness ≤2 mm, tumor size ≤2 cm, and absence of perineural invasion [8,9].

## Conclusions

Anorectal melanoma remains a diagnostic and therapeutic challenge due to its rarity and biological behavior. A high degree of suspicion should be maintained when evaluating perianal lumps, as formidable malignancies such as these may appear as benign lesions like hemorrhoids, or elusively, as areas of necrosis in a less aggressive tumor. The lack of standardized staging or management guidelines further complicates the delivery of care in this cohort of patients. Even with the best of care, prognosis remains poor, and further studies and future advancements are necessary to improve survival.
